# Supplementary material for: Machine Learning Accelerates Crystallization for Structure Determination
Source: Angew Chem Int Ed Engl. 2026 May 4;65(25):e1218503. doi: 10.1002/anie.1218503 (PMC13266958; doi:10.1002/anie.1218503)

checkCIF/PLATON report

Structure factors have been supplied for datablock(s) lcz103

THIS REPORT IS FOR GUIDANCE ONLY. IF USED AS PART OF A REVIEW PROCEDURE FOR PUBLICATION, IT SHOULD NOT REPLACE THE EXPERTISE OF AN EXPERIENCED CRYSTALLOGRAPHIC REFEREE.

No syntax errors found. CIF dictionary Interpreting this report

Datablock: lcz103

Bond precision: C-C = 0.0066 A Wavelength=1.54184

Cell:	a=19.1082(1)	b=13.4282(1)	c=17.3675(1)
	alpha=90	beta=105.436(1)	gamma=90
Temperature:	100 K		

	Calculated	Reported
Volume	4295.56(5)	4295.56(5)
Space group	P 21/c	P 1 21/c 1
Hall group	-P 2ybc	-P 2ybc
Moiety formula	C15 H3 Ag3 F18 N6, C19 H30 O5	C15 H3 Ag3 F18 N6, C19 H30 O5
Sum formula	C34 H33 Ag3 F18 N6 O5	C34 H33 Ag3 F18 N6 O5
Mr	1271.27	1271.27
Dx,g cm-3	1.966	1.966
Z	4	4
Mu (mm-1)	12.031	12.031
F000	2488.0	2488.0
F000’	2499.34	
h,k,lmax	24,17,22	24,17,21
Nref	9226	8857
Tmin,Tmax	0.124,0.186	0.695,1.000
Tmin’	0.020	

Correction method= # Reported T Limits: Tmin=0.695 Tmax=1.000
AbsCorr = MULTI-SCAN

Data completeness= 0.960 Theta(max)= 78.441

R(reflections)= 0.0396(8385) wR2(reflections)= 0.1052(8857)

S = 1.049 Npar= 599

The following ALERTS were generated. Each ALERT has the format
test-name_ALERT_alert-type_alert-level.
Click on the hyperlinks for more details of the test.

Alert level C

```
PLAT213_ALERT_2_C Atom F00S           has ADP max/min Ratio .....        3.3 prolat
PLAT213_ALERT_2_C Atom F00T           has ADP max/min Ratio .....        3.3 prolat
PLAT213_ALERT_2_C Atom F00U           has ADP max/min Ratio .....        3.2 prolat
PLAT213_ALERT_2_C Atom F00V           has ADP max/min Ratio .....        3.5 prolat
PLAT601_ALERT_2_C Unit-Cell Contains Solvent Accessible VOIDS .LE.        38 Ang**3
PLAT906_ALERT_3_C Large K Value in the Analysis of Variance ......      2.390 Check
PLAT911_ALERT_3_C Missing FCF Refl Between Thmin & STh/L=    0.600        20 Report
                2  0  0,   1  1  1,  -5  2  2,  -1  0  2,   0  0  2,   1  0  2,
                2  0  2,   1  0  4,   6  8  7,  -1 11 12,  -1 13 12, -12  5 17,
               -9  2 17, -12  2 18, -11  0 18, -11  1 18, -11  2 18, -10  1 18,
               -7  8 18,  -6  8 18,
PLAT934_ALERT_3_C Number of (Iobs-Icalc)/Sigma(W) > 10 Outliers ..         1 Check
               -7  2 20,
PLAT977_ALERT_2_C Check Negative Difference Density on H01C      .      -0.33 eA-3
```

Alert level G

```
PLAT007_ALERT_5_G Number of Unrefined Donor-H Atoms .............          1 Report
              H008
PLAT083_ALERT_2_G SHELXL Second Parameter in WGHT  Unusually Large      28.42 Why ?
PLAT142_ALERT_4_G s.u. on b - Axis Small or Missing .............    0.00010 Ang.
PLAT143_ALERT_4_G s.u. on c - Axis Small or Missing .............    0.00010 Ang.
PLAT242_ALERT_2_G Low    'MainMol' Ueq as Compared to Neighbors of       C010 Check
PLAT242_ALERT_2_G Low    'MainMol' Ueq as Compared to Neighbors of       C01E Check
PLAT242_ALERT_2_G Low    'MainMol' Ueq as Compared to Neighbors of       C01H Check
PLAT242_ALERT_2_G Low    'MainMol' Ueq as Compared to Neighbors of       C01L Check
PLAT242_ALERT_2_G Low    'MainMol' Ueq as Compared to Neighbors of       C01R Check
PLAT720_ALERT_4_G Number of Unusual/Non-Standard Labels ..........         98 Note
              Ag01    Ag02    Ag03    F004    F005    F006    F007    O008
              H008    O009    F00A    F00B    F00C    F00D    O00E    F00F
              F00G    F00H    O00I    F00J    N00K    N00L    N00M    O00N
              N00O    N00P    F00Q    N00R    F00S    F00T    F00U    F00V
              C00W    F00X    C00Y    H00Y    C00Z    H00Z    C010    C011
              C012    C013    C014    C015    C016    H016    C017    C018
              H01A    H01B    C019    C01A    C01B    C01C    C01D    C01E
              C01F    H01C    H01D    C01G    H01E    H01F    C01H    C01I
              C01J    H01G    H01H    H01I    C01K    H01J    H01K    C01L
              C01M    H01L    H01M    C01N    H01N    H01O    H01P    C01O
              H01Q    H01R    C01P    H01S    H01T    H01U    C01Q    H01V
              H01W    C01R    C01S    H01X    H01Y    C01T    H01Z    C01U
              H01     Ha
PLAT910_ALERT_3_G Missing FCF Reflection(s) Below Theta(Min)[Deg]=       4.07 Note
```

```
                1  0  0,
PLAT912_ALERT_4_G Missing # of FCF Reflections Above STh/L=  0.600        342 Note
PLAT913_ALERT_3_G Missing # of Very Strong Reflections in FCF ....          3 Note
               -5  2  2,   0  0  2,   1  0  4,
PLAT941_ALERT_3_G Average HKL Measurement Multiplicity ..........         2.6 Low
PLAT969_ALERT_5_G The 'Henn et al.' R-Factor-gap value ..........       3.275 Note
              Predicted wR2: Based on SigI**2  3.21 or SHELX Weight 10.03
PLAT978_ALERT_2_G Number C-C Bonds with Positive Residual Density.          0 Info
```

```
   0 ALERT level A = Most likely a serious problem - resolve or explain
   0 ALERT level B = A potentially serious problem, consider carefully
   9 ALERT level C = Check. Ensure it is not caused by an omission or oversight
  16 ALERT level G = General information/check it is not something unexpected

   0 ALERT type 1 CIF construction/syntax error, inconsistent or missing data
  13 ALERT type 2 Indicator that the structure model may be wrong or deficient
   6 ALERT type 3 Indicator that the structure quality may be low
   4 ALERT type 4 Improvement, methodology, query or suggestion
   2 ALERT type 5 Informative message, check
```

Validation response form

Please find below a validation response form (VRF) that can be filled in and pasted into your CIF.

```
# start Validation Reply Form
_vrf_PLAT213_lcz103
;
PROBLEM: Atom F00S            has ADP max/min Ratio .....        3.3 prolat
RESPONSE: ...
;
_vrf_PLAT601_lcz103
;
PROBLEM: Unit-Cell Contains Solvent Accessible VOIDS .LE.         38 Ang**3
RESPONSE: ...
;
_vrf_PLAT906_lcz103
;
PROBLEM: Large K Value in the Analysis of Variance ......      2.390 Check
RESPONSE: ...
;
_vrf_PLAT911_lcz103
;
PROBLEM: Missing FCF Refl Between Thmin & STh/L=    0.600         20 Report
RESPONSE: ...
;
_vrf_PLAT934_lcz103
;
PROBLEM: Number of (Iobs-Icalc)/Sigma(W) > 10 Outliers ..          1 Check
RESPONSE: ...
;
_vrf_PLAT977_lcz103
;
```

```
PROBLEM: Check Negative Difference Density on H01C       .       -0.33 eA-3
RESPONSE: ...
;
# end Validation Reply Form
```

It is advisable to attempt to resolve as many as possible of the alerts in all categories. Often the minor alerts point to easily fixed oversights, errors and omissions in your CIF or refinement strategy, so attention to these fine details can be worthwhile. It is up to the individual to critically assess their own results and, if necessary, seek expert advice.

PLATON version of 17/09/2025; check.def file version of 17/09/2025

---

duplicate check

No duplication found

Datablock lcz103 - ellipsoid plot

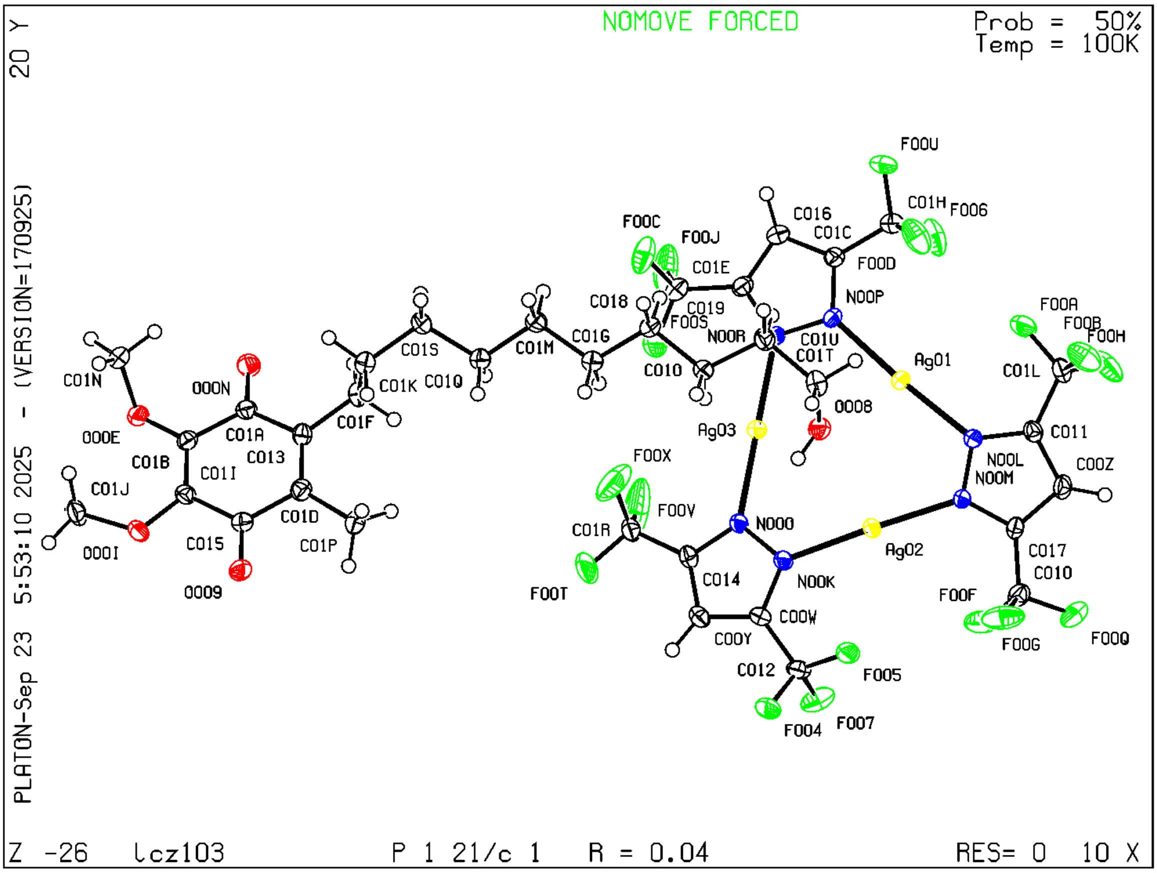

Supplement: Supplementary file 2 — Supporting File 2: anie72462‐sup‐0002‐DataFile.zip. [file ANIE-65-e1218503-s002.zip › supplementary_materials_crystallographic_data submit.zip!/cif_and_checkcif(1-114)/Ag3Pz3_103/Ag3Pz3_103_checkcif.pdf]
